# How can we assess the burden of muscle, bone and joint conditions in rural Botswana: context and methods for the MuBoJo focused ethnography

**DOI:** 10.1186/s12998-015-0056-9

**Published:** 2015-03-16

**Authors:** Maria Hondras, Corrie Myburgh, Jan Hartvigsen, Scott Haldeman, Helle Johannessen

**Affiliations:** Department of Sports Science and Clinical Biomechanics, Faculty of Health Sciences, University of Southern Denmark, Campusvej 55, Odense M, Denmark; World Spine Care, 801 North Tustin Avenue, Suite 202, Santa Ana, California, USA; Nordic Institute of Chiropractic and Clinical Biomechanics, Campusvej 55, Odense M, Denmark; Department of Neurology, University of California, Irvine, California USA; Department of Epidemiology, School of Public Health, University of California, Los Angeles, California USA; Department of Public Health, Faculty of Health Sciences, University of Southern Denmark, J.B. Winsløws Vej 9B, 5000 Odense C, Denmark

**Keywords:** Burden, Developing countries, Ethnography, Joints, Musculoskeletal system, Qualitative research

## Abstract

**Background:**

Musculoskeletal diseases are the most common causes of long-term pain and disability worldwide and a growing international public health concern. However, the everyday burden and impact of musculoskeletal conditions are not well understood, especially among people living in low- and middle-income countries in Africa. Since 2011, World Spine Care, a nongovernmental organisation, has collaborated with the Botswana Ministry of Health to open spine care centres and to conduct research. The broad aim of the **Mu**scle, **Bo**ne and **Jo**int (MuBoJo) research project is to examine the sociocultural, organisational and clinical characteristics for the burden of living with and caring for people living with musculoskeletal conditions in rural Botswana. In this paper, we describe the community context, theoretical framework, and research methods to address the project aim with a qualitative study.

**Methods/Design:**

This focused ethnography is based on eight months (November 2011, April 2013, October 2013-March 2014) of fieldwork in Botswana. The project was theoretically informed by the concepts of explanatory models of illness, social suffering, and biographical disruption. Data collection included fieldnotes, non-participant and participant observations, and informal and in-depth interviews with villagers and healthcare providers. Villager interviews were typically conducted in Setswana with an interpreter. Audio recordings were transcribed verbatim in the language spoken with Setswana contextually translated into English. Computer software supported qualitative data management. Analysis is ongoing using constant comparison and a template organising style to facilitate pattern-finding and reveal insights for the burden and care of musculoskeletal conditions.

**Discussion:**

Findings from the MuBoJo Project will document the context of musculoskeletal burden, illness beliefs, self-care behaviours, and healthcare options in a Botswana rural village. These data will inform ongoing efforts to establish spine care clinics for underserved populations in low-middle income countries and sustain these healthcare services through local providers and volunteer health professionals. This study also will generate new knowledge about the burden and impact of muscle, bone and joint disorders for cross-cultural comparisons and patient-centred interventions.

**Conclusions:**

Our systematic and transparent methodology to conduct musculoskeletal research in more than one language and in a cross-cultural setting may be useful for investigators and NGO healthcare personnel.

**Electronic supplementary material:**

The online version of this article (doi:10.1186/s12998-015-0056-9) contains supplementary material, which is available to authorized users.

## Background

Musculoskeletal disorders are the most common causes of severe, long-term pain and physical disability affecting more than a billion people across the world [1,2]. Estimates from the 2010 Global Burden of Disease (GBD) study report that musculoskeletal (MSK) disorders caused 21.3% of all years lived with disability; that back pain affects more than 632 million people worldwide; and, more than 332 million people are affected by neck pain [2]. Epidemiological studies indicate the spectrum of MSK disorders in developing countries is similar to industrialised nations, but there is speculation that the burden of disease is higher because of delays in diagnosis and lack of access to adequate healthcare facilities for effective treatment [3-6].

In Africa, health inequities and health care disparities are stark and, for most of sub-Saharan Africa, musculoskeletal health has been neglected principally due to fierce competition for scarce healthcare resources [7]. In a systematic review of back pain prevalence studies on the African continent, Louw and colleagues [8] describe that back pain affects the majority of adults in Africa, where the incidence of back pain was documented as high as 72% and the point prevalence was reported as high as 59%. Of the 27 epidemiological studies conducted in Africa and analysed in the review, none were conducted in Botswana.

Twenty-five years ago, the Botswana Ministry of Health (MoH) suggested a cooperative programme on health research that led to the collaboration between the University of Oslo and the University of Botswana led by Ingstad and colleagues, entitled “Care for the elderly – Care by the elderly” [9,10]. Building on previous work by Ingstad in the region [11-13], investigators conducted a household survey, a qualitative anthropological study and a medical study in a small rural village, Mmankgodi, located about 60 km from the capitol, Gaborone. The medical study included examination, laboratory testing and a questionnaire of 337 villagers 60 years and older. Results from the medical study indicated the most prominent health problems were related to the MSK system (446 out of 1,768 or 25% of all diagnosed conditions) with lumbar pain and neck/shoulder pain most frequently diagnosed. These and other findings from the study in Mmankgodi led the MoH to call for national data about the health and living conditions of older persons in Botswana.

Beginning in 2005, Clausen and colleagues published a series of articles [14-18] based on Botswana national household survey data collected in 1998. Clinical examinations on a subsample of *Batswana* (people of Botswana, plural)^a^ 60 years and over revealed 68% of 372 respondents reported MSK pain in two or more locations and that these painful conditions were commonly poorly managed or untreated [16].

During the past decade, there have been increased calls to refocus healthcare strategies that target non-communicable diseases, particularly in low- and middle-income countries [3,19,20]. Despite these efforts, healthcare inequities and limited resources exist in Botswana for people with muscle, bone and joint disorders. In response, a nongovernmental organisation (NGO) established two clinics and initiated research in Botswana.

### World Spine Care

In 2008, World Spine Care (WSC) was founded by co-author SH as a NGO to help people with spinal disorders in underserved communities throughout the world. The WSC tripartite mission is grounded in the clinical, educational, and research domains aiming to develop a low cost model of care for primary spine care clinicians, educate local healthcare workers and patients, and conduct research. Two of the authors (MH and JH) volunteered to serve on the WSC research team in 2010 and 2011, respectively.

By June 2011, WSC and the government of the Republic of Botswana represented by the MoH signed the Memorandum of Understanding to establish spine care centres in Botswana rural communities. The initial target areas are located in the Central District, where WSC aims to develop sustainable capacity for effective interdisciplinary spinal care in Village Shoshong and the Mahalapye regional health district. WSC volunteers include primary spine care clinicians (chiropractors and physiotherapists) and, in the future, specialty care providers (rheumatologists, orthopaedic surgeons, neurologists and radiologists) who serve various tours of service ranging from several weeks to one or more years. One example of the MoH and WSC reaching for sustainable capacity is that in 2014, the first two *Motswana*, both with more than 20 years experience as nurses in Botswana, began their professional training in chiropractic programmes in North America. By 2019, upon completion of these programmes and additional training with WSC, they will assume management and operations roles for the WSC clinics in Shoshong and Mahalapye.

As a NGO devoted to spine care in Botswana, WSC has long term goals, in part, to: a) conduct epidemiological studies; b) establish education programmes; c) ensure interaction of health professionals; d) implement a front-line worker training programme; e) establish ongoing and sustainable spine care; and, f) create relevant ways to assess the impact of WSC education and treatment programmes. With these ambitious goals rooted in a western healthcare paradigm and to inform the longer term goals for WSC, investigators agreed that one way forward was to begin with a qualitative investigation to better understand the burden of and care for MSK conditions in Shoshong.

### Project aims

An important foundation for the optimal management of MSK conditions is an in-depth understanding of the everyday life burden of people suffering from muscle, bone and joint disorders. Although ‘counting people’ with conditions provides important prevalence information, a qualitative approach is suitable to explore the meanings that people attribute to particular life events, including health and illness [21]. Explicating the process of health care that villagers currently use, as well as their hopes and expectations about future care for MSK conditions will be important to develop and sustain health education and service models. In this way, we might “set the needs of our patients in their appropriate context (and begin) to understand why patients and practitioners do what they do and how we might (directly and/or indirectly) influence these processes to improve clinical outcomes” [22].

The broad aim of this qualitative research project is to examine the cultural, social, clinical and organisational characteristics for the burden of living with and caring for people living with **Mu**scle, **Bo**ne and **Jo**int (MuBoJo) conditions in a Botswana rural setting – Village Shoshong. In essence, we want to understand the concept of MuBoJo societal and individual burden to inform socioculturally relevant strategies to prevent and manage muscle, bone and joint conditions.

### Theoretical framework

With the broad brush aim to explicate the burden of and care for MuBoJo conditions in Shoshong, we drew on theoretical perspectives from medical anthropology, sociology and transcultural psychology. Craig et.al. note that theoretically driven, “qualitative research tools to draw out illness narratives … have been used to: gather data for socio-cultural analysis, improve communication between patients and providers in clinical settings, help develop health-related interventions, and evaluate health programmes and therapeutic regimens” [23].

Our research framework was inspired by Arthur Kleinman’s [24] notion that “(H)ealth, illness, and health care-related aspects of societies are articulated as cultural systems.” He posited that “(T)he health care system is a concept, not an entity; it is a conceptual model held by the researcher” [25]. His model for the ‘inner structure of health care systems’ – comprised of popular, folk and professional sectors – guides our aims to study how villagers and healthcare practitioners “in a particular social setting *think* about health care” [25] [emphasis in original]. We believe this framework is important to examine how public health approaches for MuBoJo care intersect and integrate into the everyday lives and current healthcare seeking behaviours of Shoshong villagers.

Kleinman’s work from the 1960s and 1970s regarded explanatory models (EMs) as “the notions about an episode of sickness and its treatment that are employed by all those engaged in the clinical process” [25]. Perhaps unsurprising to healthcare providers, Kleinman’s EMs sought to explain five elements for illness episodes including: etiology; time and mode of onset of symptoms; pathophysiology; course of sickness (including both degree of severity and type of sick role – acute, chronic, impaired, etc.); and treatment. He cautioned that EMs for discrete illness episodes be distinguished from more general beliefs about sickness and healthcare.

Almost two decades later, Kleinman wrote that he intended “the explanatory models technique to be a device that would privilege meanings, especially the voices of patients and families, and that would design respect for difference” [26]. It is this perspective that guides our attention to understand life lived with MuBoJo conditions and give voice to rural *Batswana* who suffer from and care for both themselves and for others who live with MuBoJo troubles.

We also draw on Kleinman’s [26] concept of ‘social suffering’ and Michael Bury’s [27] notion of ‘biographical disruption’ to better understand the lived experiences, meaning and context for the burden of MuBoJo conditions among Shoshong villagers. From these perspectives, we will follow the thread that chronic conditions (such as arthritis) produce not only physical and physiologic disabilities, but that social identities and life trajectories also are disrupted and that the burden of illness underlying MuBoJo conditions remains significant [28]. We also will examine the intersubjectivity and interconnectedness [26] of cultural influences that best support (or reject) the spine care model that is principally based on a biomedical approach to care. As Bury suggests, “meaning and context in chronic illness cannot be easily separated” [28]. Thus, empirical evidence will be important to assess the need and population dimensions for MuBoJo burden and care in this rural developing country setting.

The foregoing perspectives do not preclude other theoretical lenses to illuminate study findings during analysis. Certainly we will make essential links between the literature, theory and method when our analysis is complete and situated in a broader context [29]. But at the outset of the MuBoJo project, we believed that illness narratives and explanatory models would be important to explicate as we partner with villagers, healthcare personnel and government to reduce the burden of MuBoJo troubles and improve quality of life.

## Methods

### Study design

The MuBoJo project is a focused ethnography. Researchers have long used ethnography as a comparative method to investigate patterns of human behaviour and cognition through observations and interactions in natural settings [30]. In short, ethnography is “the work of describing culture” [31,32]. Historically, ethnographers engaged in long-term field studies, most lasting a year or longer, with the goal of comprehensively depicting a cultural group.

Focused ethnography is a methodology that emphasises anthropological theory and methods while limiting the scope and duration of fieldwork to a specific program-relevant research problem and is “often used to determine ways to improve care and care processes” [32]. One of the virtues of ethnographic research is “that it remains flexible and responsive to local circumstances” [33]. Because we wanted to explore the meaning of a set of related concepts about the burden and care of MuBoJo problems, alongside the temporal relationships of establishing spine care centres in rural Botswana and conducting research during a three-year doctoral programme, we designed a focused ethnography.

Although *a priori* research hypotheses are not appropriate for this methodology, research questions adapted from Kleinman [25] guided development of the project aim, methods and analytic concepts. These included:

- What are the meaning contexts of burden and muscle, bone and joint conditions in Shoshong?
- What are the paths people travel when they have muscle, bone or joint troubles?
- What are the culture-specific and universal characteristics of caring for MuBoJo conditions in Shoshong?

### Setting

#### Botswana

Botswana is a land-locked country in the southern part of the African continent, bordered by South Africa to the south and southeast, Namibia to the west and north, and Zimbabwe to the northeast [see Additional file 1 for a map of Botswana]. The Kalahari Desert covers almost 70% of the land such that with a population just over two million people [34], Botswana is one of the most sparsely populated countries in the world. The Okavango Delta lies in the north and is the world’s largest inland delta. Most of the population lives along the eastern border of the country with approximately 40% living in rural areas. Botswana is comparable in size to France and slightly smaller than Texas in the USA.

English is the official language of the government, business and education sectors, however at least 80% of the people speak Setswana, with smaller proportions who speak Kalanga (8%) and Sekgalagadi (3%) [34]. Fifty-one percent of *Batswana* are female [34]. The average life expectancy is 53 years for both men and women [35], although with the reduction in infant mortality rate and the government’s increased access to antiretroviral drugs, life spans are expected to increase in the near future.

The Botswana healthcare system includes public (governmental), private for-profit, private non-profit and traditional medicine practice, with 98% of health facilities operating in the public sector [36]. Healthcare services are decentralized to the district level; delivery is based on the primary health care model; and until 2011, oversight was provided by Village Development Committees (personal communication, participant 36). The MoH now oversees health services in the country. There are 14 district hospitals in country, more than 200 health clinics, at least 330 health posts, and almost 850 mobile posts. Most of the population lives within 8 km (about 5 miles) of a health facility. At present, health systems principally provide care for acute episodic conditions and do not tend to chronic care needs, particularly for the rapidly growing aged population.

#### Village Shoshong

Shoshong is a rural village of about 10,000 people in the Central District of Botswana, just north of the Tropic of Capricorn and approximately 250 km (150 miles) north of the country’s capital, Gaborone (more than 230,00 people) [34]. There are three wards in Shoshong: Phaleng, Bokaa and Kgamane. Wards are generally areas where residences are clustered around the *kgosi’s* (chief’s) residence and ward *kgotla* (traditional village meeting place). Phaleng and Bokaa are the largest Shoshong wards, each with roughly 35 sub-wards (personal communication, A. Plant). Shoshong is fairly typical of a large village in Botswana where people maintain a ‘three-home system’ and travel from the village to the lands and to the cattle-posts [37]. There is one tarred road leading into Shoshong from the east; this road bifurcates at the bus rank into two tarred roads that end up parallel to each other as one travels west to the next major road (about 10 km) – known simply as ‘the road to Gaborone’ heading south and ‘the road to Serowe’ to the north. The remainder of the 700 square miles (1813 square kilometers) that make up Shoshong include rugged terrain, corrugated sandy roads, thick bush, and dry, rocky, red earth. During the rainy season, many corrugated roads are impassable and a significant proportion of villagers are unable to receive aid or emergency services.

#### Study access

Gaining access to Shoshong for this focused ethnography is epitomised by Hammersley and Atkinson [33], where “(s)ometimes the setting itself comes first – an opportunity arises to investigate an interesting setting or group of people; and foreshadowed problems spring from the nature of that setting”. Certainly, it can be argued that, we harnessed investigator interest in MSK burden with collaborative relationships amongst NGO and university personnel to develop an ‘opportunistic research strategy’ [38] that would best serve villagers in rural Botswana.

### Participant recruitment

People living in and around Shoshong who suffered from or cared for people suffering from muscle, bone and joint conditions were eligible for this study, including villagers who had and had not attended the NGO Clinic, health professionals and staff from two village clinics and one health post, and traditional healers. We used purposive, opportunistic and snowball sampling to invite participants regardless of age, gender, social status or physical condition; in this way, we sought information-rich cases to illuminate the research aims under study [39].

Villagers were approached while the researcher (MH) was ‘hanging out’ in the village and during several scheduled *kgotla* gatherings. Other participants were self-selected during participant observations, informal interviews, and by word of mouth from *kgotla* presentations, clinic staff meetings, and community events where permission was granted to ask for volunteers. A color brochure [Additional file 2: Setswana and Additional file 3: English] was available at the three main *kgotlas,* village clinics, and library, and was offered to potential participants when MH engaged villagers in conversations during her stay*.* The brochure aimed to position the researcher as researcher during fieldwork, rather than as a NGO healthcare provider.

All healthcare providers and staff who worked at the NGO clinic during the time of fieldwork were invited to participate. We also invited nurses, health education assistants and health care auxiliaries working in the two Shoshong primary care clinics and one health post to participate in the MuBoJo project.

### Ethical considerations

The Botswana Ministry of Health, Health Research Development Committee granted ethics approval (HRDC 00735) for this project, as well as approval for our continuing review request for data analysis. The Botswana HRDC follows WHO ethical review standards [40] and in 2012 Botswana had “the highest proportion of Research Ethics Committees (five) to population and of number of people trained (four) to population” in 25 African countries evaluated, despite its ranking seventh and eighth on those parameters, respectively [41]. In addition, we obtained verbal permission to conduct the study in Shoshong from the traditional authorities, the *dikgosi*, from the three main wards.

For transparency and to assure village leaders that we were treating people in a respectful way, we provided the detailed informed consent document (ICD) in Setswana and English to the *dikgosi,* the Shoshong Clinic head matron and senior health education assistant, and all villagers who requested these details [Additional file 4: Setswana and Additional file 5: English]. The ICD outlined the study purpose, procedures, risks and benefits, methods to ensure privacy and confidentiality, voluntary nature of participation and the right to withdraw from the study at any time. These details follow Western bioethical principles on which the Botswana MoH application guidelines are framed. For in-depth interviews, the researcher and interpreter administered consent orally at the time of the interview and participants signed the Statement of Consent [Additional file 6: Setswana and Additional file 7: English], which was a one-page summary about the voluntary nature of participation; that they could stop the interview at any time and not need to give a reason; that detailed ICDs were freely available for review; and they could ask questions at any time. There was also a selection where the participant did or did not give permission for audio recording the interview. For photographs, we obtained separate (recorded) verbal and/or written permission [Additional file 8: Setswana and Additional file 9: English] for use in scientific presentations and publications related to the MuBoJo project. We prepared child assent ICDs for potential participants under 18 years; however, no children participated in the project.

Because the interpreters and transcriptionists may be acquaintances of study participants, MH conducted individual training sessions about the importance of confidentiality, the protection of human participants, and complying with secure data transfer protocols for interview transcripts. MH required all Botswana-based project personnel to complete the online certification course from the U.S. National Institutes of Health Office of Extramural Research entitled ‘Protecting Human Research Participants’ [42]; MH has completed annual recertification from this program for the past 10 years. At the end of the online course, personnel submitted their certificate of completion for project files and met with MH to discuss course content.

### Data collection

Earlier visits to Botswana in late 2011 and early 2013 for five and four weeks, respectively, afforded opportunities to: establish relationships with government officials and health care personnel; seek approval from traditional authorities to conduct research in Shoshong; tour existing healthcare facilities and develop relationships with local healthcare providers and WSC clinic personnel; build local capacity with *Batswana* interested in assisting with the project; prepare forward and backward translations of text documents; and, pre-test the interview and transcription processes in two languages.

Ethnographic material is drawn primarily from six months of fieldwork during October 2013 to March 2014. Routine efforts to generate daily fieldnotes, transcribe and translate digital recordings, index and file material, write memos and reflexive notes were demanding, time intensive activities [33]. Verbal fieldnotes were habitually captured in a digital voice recorder, particularly during the 40 km commute between the study site and NGO accommodations or during the two-hour commute to Gaborone to train and work with transcriptionists, transport NGO volunteers to/from the airport, and build relations with university-based faculty.

The researcher engaged in non-participant observation in natural settings where people interact with one another, including *kgotlas,* bus rank, shopping areas, post office, and clinic waiting rooms. Participant observations included daily work routines in people’s compounds, at the lands and at cattle posts. In this way, MH was able to interact and participate with people during their work and leisure time to observe and talk with them about what they were doing, thinking and saying [43] in relation to muscle, bone and joint health. These data enhanced the content of interviews and some observations led to in-depth interviews or photographs.

In depth interviews were conducted in settings convenient and comfortable for participants and, with two exceptions, were audio recorded. With few exceptions, villager interviews were conducted in Setswana with an interpreter. Interviews with healthcare providers were typically conducted in English.

Fifty-five interviews were conducted with 34 villager participants in April-May 2013 and between October 2013 and March 2014. Twenty-four villagers were interviewed once, five were interviewed twice, two interviewed three times, and three villagers were interviewed four, five and six times, respectively. Ten pre-test interviews ranged from 10–35 minutes each with an average interview duration of 20 minutes. The remaining 45 interviews ranged from 30–90 minutes with an average duration of 60 minutes. Of the 34 villager participants, 25 were women and nine were men. The median age of participants was 57 (range = 20-97).

Fifteen interviews were conducted with 14 healthcare providers; one provider was interviewed twice, approximately ten months between interviews. Providers were licensed, certified or apprenticed as a chiropractor, healthcare auxiliary, health education assistant, nurse or nurse/midwife, osteopath or traditional healer. Of the 14 providers, 10 were women and four were men. Ten providers were *Motswana* and one each were from France, South Africa, Switzerland and the United States. Age was collected in 10 year increments such that four providers were in their 20s, four in their 30s, five in their 50s, and one more than age 60. On average, provider interviews lasted for one hour.

### Language strategies

Given the opportunistic research setting with researchers and NGO volunteers who do not speak Setswana and the aims of this research to reveal the everyday burdens of MuBoJo conditions, we designed several strategies to work with translators (for written language), interpreters (for spoken language), and transcriptionists (to transcribe audio recorded Setswana and provide contextual translations in English).

#### Written language

During MH’s earlier visits to Botswana, along with relationship building between WSC personnel and people in Gaborone and the Central District of Botswana, two women agreed to provide forward translations (English to Setswana) for text documents. Both women are Botswana nationals, fluent in both languages with Setswana their mother tongue. One woman in her 50s has diplomas in general nursing and midwifery, a BS in health sciences, and more than 20 years experience in the public health arena, including local, national and regional community health efforts; she currently lives in Mahalapye (about 40 km from Shoshong). The other woman is in her 40s with a BA in Humanities and post-graduate certificates in AIDS management, monitoring and evaluation; she has served as a field coordinator for over ten years with a consultant’s agency based in Gaborone that provides project management services in Botswana. The translators do not know one another and both prepared independent forward translations for project text documents, including the informed consent document, photograph permission form, information sheet, human placard, and semi-structured interview guides.

Next, two Shoshong villagers who were not involved with forward translations conducted independent backward translations. These villagers also happened to be women, both in their 20s, and both having completed senior secondary schooling (high school equivalent in the US) in Botswana. At the time, these young woman were volunteers at the NGO clinic, serving the capacity of healthcare auxiliary (clinic staff).

Through this process, one set of forward translations emerged as the least complex translation and best suited for villager understanding. Back translations allowed us to simplify content in both languages to adapt textual information to suit the culture [44]. Through discussions with three of the four translators, we agreed that the information sheet was cumbersome with far too much detail; this was completely revised and designed as the color brochure [Additional file 2: Setswana and Additional file 3: English] to present simple and consistent messages about the research project.

#### Spoken language

The translator who lives in Mahalapye was enthusiastic to continue work with the MuBoJo project and offered her services as an interpreter. During the pre-test phase in April 2013 and after text document translations were complete, MH and the interpreter spent one week working together. We discussed issues of confidentiality, semi-structured and open ended questions, and conducting interviews in two languages. We pre-tested the interview process by conducting 11 patient and provider interviews together; interviews were audio recorded and the average duration was 20 minutes. On-going dialogue between MH and the interpreter focused on: the ideas expressed with interview questions, refining our approach so that questions had relevance in the local setting [44], and techniques to maximise the richness and depth of the data obtained. We reconnected in October 2013 and worked together for six months during the primary data collection phase, although there were several days during each month of fieldwork when the interpreter was unavailable. Thus, a second interpreter was hired during the early days of fieldwork. This man is a *Motswana* in his 30s, whose first language is Kalanga and also speaks Setswana and English. He has served as an interpreter for staff at the WSC clinics in Shoshong and Mahalapye at various times over the past three years.

#### Transcribed language: data transcriptions and translations

Before departure in April 2013 MH worked with two independent persons (one female; one male) who expressed interest to serve as transcriptionists for interviews conducted in Setswana. The woman from Gaborone had prepared one set of forward translations for text documents. A serendipitous conversation with a key informant led to a young man in his 20s from Shoshong who also expressed interest in transcription work. This man spent his formative years in Shoshong and his teenage years in Gaborone before entering university in Malaysia; he has a BA in Mass Communication and Broadcasting.

One-on-one training with each potential transcriptionist included discussion about issues of confidentiality, secure data transfer protocols, and pre-testing the transcription process. We created a transcription guide [Additional file 10] with examples of notation preferences, particularly for inaudible sections of audio files and conversations with emotional content [45]. MH transcribed English from two of the pre-test interview audio recordings and inserted placeholders when Setswana was spoken. Transcriptionists agreed to provide verbatim Setswana transcription and contextual English translation of dialogue. All parties agreed to pre-test the transcription process during the next month and then revisit negotiations for future work.

One transcriptionist, the Shoshong villager, completed two transcripts within two weeks. Several attempts to reach the second potential transcriptionist were unsuccessful by the time the MoH ethics application was submitted in June 2013. Hence, one transcriptionist was listed on the ethics application and we acknowledged that we would need several transcriptionists to keep pace with interviews during fieldwork. We were optimistic that university graduate students would be interested in the work once MH was on the ground for an extended stay.

During fieldwork that commenced in October 2013, MH or a professional transcriptionist (Way With Words Ltd., London) prepared verbatim English transcriptions and inserted placeholders for Setswana spoken during the interviews. MH relistened to entire audio recordings to review and correct vendor-prepared English transcripts before passing documents to Botswana-based personnel for verbatim Setswana transcription and contextual English translation. As predicted, one *Motswana* transcriptionist could not keep pace with the volume of in-depth interview data collected during fieldwork. Consequently, five *Motswana* were trained and hired over the course of six months. In the end, three of these transcriptionists only transcribed seven interviews, four of which were unusable and re-assigned. Of 56 audio recordings with two languages, two *Motswana* prepared 22 and 31 transcripts, respectively.

### Researcher team profiles

Regardless of the analytic approaches we adopt for this research, personal and professional identities and interests will inevitably shape how we describe and interpret the data [46,47]. Harnessing these resources was important for planning and conducting the MuBoJo project; acknowledging the subjectivity of researchers is important for analysis. All five authors are white and from upper middle class backgrounds.

MH is a second generation Greek-American born in the eastern United States. Her manual therapy interests date back to the early 1970s when she practiced massage therapy. She received a graduate degree in chiropractic from the National College of Chiropractic (now the National University of Health Sciences) in 1989. She earned a master’s degree in public health (epidemiology) in 1993 and maintained a limited home-office chiropractic practice for 15 years. She spent 23 years teaching critical appraisal of the literature and fundamentals of epidemiology in chiropractic curricula, graduate and post-graduate programs in the US. Since 1990, she has been involved with the design and conduct of clinical trials in manual therapy, particularly chiropractic. By 2009, her research interests shifted to qualitative research. Since then, she served as co-facilitator for several focus groups, in which she assisted with the analysis and publication of the work [48], and is engaged in analysis of semi-structured interviews with participants from several clinical trials. In 2012 she enrolled in a doctoral programme at the University of Southern Denmark (SDU) Faculty of Health Sciences. She embarked on the MuBoJo project with a passion to provide empirical work for the burden and care of MSK disorders in underserved communities.

CM is a chiropractor who received his training at Technikon Natal (now Durban University of Technology) and a doctorally-prepared social scientist (University of Stellenbosch) who was born, raised and educated in South Africa. After one year in private practice, he assumed several lecture and administrative roles in the chiropractic department at Durban University of Technology. He joined SDU as a post-doctoral fellow in 2006 and is now an associate professor in the Clinical Biomechanics Research Unit. He remains clinically active in private and public settings. His research interests are in the politics and legislation of chiropractic, as well as in the notion of recovery from musculoskeletal conditions.

JH is a Danish chiropractor who received his chiropractic degree from Palmer College of Chiropractic (PCC) in Davenport, Iowa and an epidemiologist who trained at SDU. He is professor and head of the SDU Clinical Biomechanics Research Unit; he is also head of the SDU Graduate Programme for Physical Activity and Musculoskeletal Health. His research focus has been on spinal and MSK pain in the population and he has been active in Danish national and international task forces and health technology assessment groups for spine pain, traumatic brain injury and evaluation of MSK research.

SH was born in Canada but spent his formative years in South Africa where he completed his undergraduate and master’s degree. He completed professional training in North America, obtaining his chiropractic degree from PCC, and his doctoral (neurophysiology) and medical degrees from the University of British Columbia. He is a US board certified neurologist, a fellow of the Royal College of Physicians of Canada, and currently in clinical practice. He also is the World Spine Care president. His current clinical and research foci are aimed at providing evidence-based, culturally integrated prevention, assessment and treatment of spinal disorders in the developing world. He has long supported interprofessional and multidisciplinary approaches to care for people with spinal disorders.

HJ is a professor and anthropologist having received her doctoral training at the University of Copenhagen. She has 30 years experience in academia at the intersection between anthropology and health sciences. In addition to medical anthropology, her research interests include qualitative methodology in health research, medical pluralism, and embodiment of political structures. As a native Dane, her professional trajectory has included multiple international collaborations with complementary and alternative medicine investigators.

### Data analysis

On-going data analysis is a prominent feature of ethnographic methods, whereby dimensions important to participants unfold during fieldwork and create iterative loops through various phases of data collection. Our iterative analytic process is guided by the ‘grounded theorizing’ approach described by Hammersley and Atkinson [33]. With this approach, there is no formula or recipe for ethnographic data analysis, but it is important to recognise that data management and manipulation are not enough; rather “(D)ata are materials to think with [33]”.

While analysis continues at the time of this writing, initial stages included reading and re-reading interview transcripts and fieldnotes, and generating memos for concepts that made sense of the data. Following close reading of textual data and initial categorical coding, MH creates substantive codes for each topic and groups these into major themes. She uses constant comparative methods to identify similarities and differences within and across cases, returning to the raw data iteratively to review categories and themes, clarify meaning, and reflect on patterns and connections emerging from the data [33]. When in the field, she consulted with her interpreters and key informants to ensure that her understanding of the cultural context was the villager’s understanding of cultural context. Although technological challenges in rural Botswana thwarted communication with international colleagues during fieldwork, upon returning from the field, MH regularly reviews and discusses data analysis and interpretation with co-authors.

This structured approach or template organizing style [49] of analysis aims to facilitate pattern finding to reveal insights and meaning about the burden of musculoskeletal conditions. We are using NVivo 10 (QSR International, Victoria, Australia) computer software for data storage and retrieval from a large volume of fieldnotes, transcripts, and memos, to assemble these data in one place for the interpretive process. The software allows us to organise codes derived from the data and merge codes into larger categories and themes as the analysis progresses.

Notwithstanding the value of coding these data to identify similarities and differences between cases for the research questions related to the paths traveled to attend to MuBoJo conditions and potential comparisons of culture-specific and universal characteristics of MuBoJo conditions, we are sensitised to the notion that the meaning contexts may be non-itemizable [50]. Drawing on Biernacki’s argument “that coding frustrates retrieval of the nuanced meanings that explain action,” we also believe the immersion/crystallization (I/C) analytic technique will be valuable to unpack MuBoJo burden from these data. I/C was originally coined by Crabtree and Miller [51] and further elucidated by Borkan [52] and demands ‘personal immersion in the data, by repeated reading of texts, until insights become apparent’ [53]. Findings and interpretations will be presented and discussed among research team members to reach consensus about explanatory models for the burden of living with and caring for others with MuBoJo conditions in Shoshong.

### Data quality

Given that the researcher does not speak Setswana and given that most elders in Shoshong speak little English, we used rigorous and transparent methods in our approach to gather data. We did not impose preconceived themes on the data collected. Reflexive reading will locate the researcher as part of the data generated and will seek to explore her role and perspective in the process of generating and interpreting the data.

We aimed for credibility and confirmability in several ways. First, we developed an early familiarity with village culture [54] during visits in 2011 and 2013 before designing the study and entering the field for primary data collection. Second, we provide in this report, a detailed description of the context and methods used with this focused ethnography. Next, we are in the midst of prolonged engagement with the texts and coding process, including debriefings with experienced research mentors. For fieldnotes and transcripts generated during fieldwork, the researcher consulted key informants fluent in both languages to reduce misinterpretations of the data. Further on, we sought a wide range of informants to confirm or disconfirm patterns that emerged during data collection. Finally, on-going reflections about the nature of this journey with villagers adds to the contextual framework for analysis and for future World Spine Care work in Botswana. We have no reason to believe that Shoshong villagers, who suffer from or care for others living with muscle, bone and joint conditions, differ from villagers in other rural Botswana communities. Readers will need to determine the transferability of findings to other settings.

## Discussion

To our knowledge, this is the first report of the methodological features for an ethnographic study in Botswana. We provide details about the study aims; the contextual relationships between a non-governmental organisation, World Spine Care, and the Botswana Ministry of Health; profiles for an international, multidisciplinary investigative team; and, the methods used to conduct the MuBoJo project. We anticipate completing data analysis by mid-2015, with a report of findings to the Botswana Ministry of Health, Shoshong *dikgosi* and villagers to follow, along with reports to funders and open literature publications.

Several aspects of the methods for this qualitative research effort merit discussion. Our study methodology has several strengths. We negotiated access and established rapport with villagers during several visits to the site, including first stops with the traditional authorities, the *dikgosi,* at the start of each visit. Even with first contact and our request to conduct research by hanging out in the village and talking with villagers in their homes, the immediate response from village chiefs was, “when will you begin? You are welcome in our village”. With successive visits, the researcher was warmly welcomed and introduced to village elders who facilitated access within the village wards and who literally passed the word that “this woman wants to speak with you about your *mesifa, marapo le ditokololo* (muscle, bone and joint) troubles”. This purposeful, opportunistic and snowball sampling provided depth and breadth to the number and quality of casual conversations, in-depth interviews, and participant observations in village homes, at the lands and at the cattle posts.

For transparency in data collection, we provide detailed methods for engaging the services of translators (for written text), interpreters (for spoken language) and transcriptionists (for transcribed language to be used in analysis) [55,56]. These methods were rigorous and practical in this setting. We also required *Motswana* study personnel to complete a standardized training for the protection of human participants [42] and were flexible to provide consistent training methods with multiple people when each was ready to engage in the work.

The extended time for fieldwork allowed investigators to nurture community buy-in for the MuBoJo project, conduct follow-up interviews with many participants, and establish collaborative relationships with healthcare personnel at the village clinics, district hospital and district health management team (40 km away), and one health post (80 km from the village) who provide services and care for villagers. Time in the field also afforded the opportunity for reflexivity [33,43] about the ethnographic methods used for the MuBoJo project, to think critically about researcher roles and relationships developed [57], and make resolute plans to uncover negative cases or disconfirming evidence [58] of early analytic concepts revealed from interviews and fieldnotes. In this report, we identified personal and professional characteristics of the investigative team [59], to make transparent the lenses and experience that we, as investigators, will bring to the analysis and interpretation of the data.

Our study is not without limitations. None of the investigators speak Setswana and the lead ethnographer typically relied on interpreters to conduct interviews and engage in participant observations. However, we provide strategies to minimise language limitations with a determined pursuit to engage local *Batswana* to assist with the MuBoJo project and transparent methods to prepare and interpret written, spoken and transcribed texts. For written texts used with participants, we implemented methods that were most practical in this setting. Our minimum standard was to generate two forward and two backward translations with four independent *Motswana*, followed by revisions, pre-testing and submission of revised documents to the Ministry of Health for ethics approval. Ideally, we would have included an expert review committee to reach consensus on discrepancies and determine semantic, idiomatic, experiential and conceptual equivalence [60]. However these standards are recommended for the development of self-report outcomes measures; in the MuBoJo project, we did not include these measures. Rather, we prepared Setswana and English versions of the recruitment brochure, informed consent document, photograph permission form, a human placard with identification of body parts, and semi-structured interview questions.

The time required to prepare written text took five months, with the most efficient use of time when the principal investigator was in country working with local collaborators to facilitate work products. Although advances in communications technologies allow for long distance collaborations, a digital divide remains between North American and European countries and countries in the rest of the world, and in particular, for people living in African countries [61-63]. For example, two of the four translators used paper and pencil to prepare documents, as they did not have access to a computer. Of the two who had computer access, neither had a stable internet connection for file transfers. In addition, functioning internet and telephone connection, and even electricity, was not always available to the investigative team when in Botswana. NGOs conducting research in underserved countries must plan ample time to prepare documents in the local language, preferably with investigators on the ground, to work hand-in-hand with the local project team.

For spoken and transcribed language, and in hindsight with transcripts now complete, we can see that interpreters synthesised participant dialogue during the interviews as best they could, but sometimes did not give a comprehensive account of the villager’s voice. Analysis of transcribed text will reveal the comprehensiveness of the conversations and identify areas of inquiry for future visits and further in-depth interviews with villagers. Lessons learned working with interpreters *during* interviews will be discussed in more detail in a future publication, to explore the “social location of the interpreter and, along with the researcher and interviewee, their impact upon the construction of the interview accounts” [64]. In brief, our team aimed to “carry out interviews *with,* rather than *through,* interpreters” [64] where we acknowledged the co-construction of data between interview participants, the interpreter and the researcher [56,65].

To enhance data quality during fieldwork, the principal investigator relistened to audio recordings of completed transcripts and worked with transcriptionists to ensure all sections where Setswana was spoken were included in the transcribed text. She also conducted follow-up interviews with some participants to pursue more comprehensive lines of inquiry. Looking forward, solidifying collaborative relationships with *Motswana* academics in health sciences will enhance interpretations and synthesis of findings. Perhaps timing and funding will always be considerations, but several *Motswana* faculty and staff from the University of Botswana were interested in contributing to the MuBoJo project, but unable to commit their time during our fieldwork.

There were challenges to hiring and retaining transcriptionists for the project and for ‘real time’ transcript preparation such that ongoing analysis could dictate potential areas of inquiry related to the burden and care of MuBoJo conditions for all participants. English transcription took approximately six hours for every hour of recorded interview. For Setswana transcriptions with contextual English translations, project personnel spent at least 12–15 hours for every hour of recorded interview and we have 70 hours of audio recorded interviews. Purchasing services for translators, interpreters and transcriptionists requires time, money, and flexibility. This is important for NGOs establishing clinics in countries where volunteers do not speak the language and where resources are limited and devoted to clinical care, as opposed to supporting qualitative research projects with observations, interviews and focus groups.

Finally, eight months of fieldwork can be viewed as both short and long term engagement. Given the three-year doctoral programme and the motivation to unearth practical and culturally relevant findings for the broader goals of the NGO, eight months provided a considerable amount of data for analysis. The initial protocol designated two months for primary data collection. However, after the first two visits, approximately one month each, we realised another two months would only provide superficial examination of the burden and care for MSK conditions. Six months in the field allowed MH to nurture relationships with traditional authorities and villagers; position herself as researcher, rather than as a NGO volunteer in the clinic; work closely with *Motswana* project personnel to enhance the data collection processes; and, implement the MuBoJo project, including follow-up interviews with many participants. Ethnographic fieldwork for a longer time will permit the researcher to learn the language.

Focused ethnography is a research approach that includes intensive efforts to examine research questions that “require understanding the complex social and cultural contexts of people’s lives” [66]. The everyday burdens of living with and caring for people living with musculoskeletal conditions are not well represented in the literature, particularly in low- and middle-income countries. With this focused ethnography we want to give voice to rural *Batswana* who suffer MuBoJo conditions and illuminate culturally sensitive paths for collaboration to provide healthcare services that reduce everyday life burden and improve quality of life. Building multidisciplinary and multinational sustainable partnerships is integral to reduce musculoskeletal health inequities in developing countries [7,67], as well as in underserved areas of industrialised nations. We hope this work can inform clinicians about the research process for conducting ethnographic research in the communities they serve. We are optimistic about identifying what is important to Shoshong villagers and how we can sustain World Spine Care initiatives that prove valuable.

## Conclusions

We report the context and methodology for a focused ethnography to examine the burden of living with and caring for people living with muscle, bone and joint conditions in a rural village in Botswana. We believe that our experiences to systematically address the methodological challenges to conduct research, in more than one language and in a setting with sociocultural contexts far removed from investigators and NGO healthcare personnel, should be transparent and freely available to others.

## Endnote

^a^Julie Livingston [68] provides a lovely, succinct description for the language terms we use in this project: “Setswana, like all Bantu languages, classifies nouns through a system of prefixes. *Setswana* refers to the language and culture of the Tswana ethnicity. *Motswana* is a single Tswana person, *Batswana* are multiple Tswana persons, and *Botswana* is the collective noun for all Tswana people and hence the name for the modern nation”.

## Additional files

Additional file 1:
**Map of Botswana showing the study site, Shoshong (circled in red).** Royalty free map purchased from World Trade Press^©^ on 27 September 2014. Additional file 2:
**MuBoJo brochure, Setswana.**
Additional file 3:
**MuBoJo brochure, English.**
Additional file 4:
**Informed Consent Document, Setswana.**
Additional file 5:
**Informed Consent Document, English.**
Additional file 6:
**Statement of Consent, Setswana.**
Additional file 7:
**Statement of Consent, English.**
Additional file 8:
**Photograph permission, Setswana.**
Additional file 9:
**Photograph permission, English.**
Additional file 10:
**Transcription guide.**

## Abbreviations

EMs: Explanatory models
GBD: Global burden of disease
ICD: Informed consent document
MoH: Ministry of Health
MSK: Musculoskeletal
MuBoJo: Muscle, bone and joint
NGO: Nongovernmental organisation
SDU: University of Southern Denmark
WSC: World Spine Care
