# Supplementary figures and images for: How can we assess the burden of muscle, bone and joint conditions in rural Botswana: context and methods for the MuBoJo focused ethnography
Source: Chiropr Man Therap. 2015 Mar 16;23:11. doi: 10.1186/s12998-015-0056-9 (PMC4361207; doi:10.1186/s12998-015-0056-9)

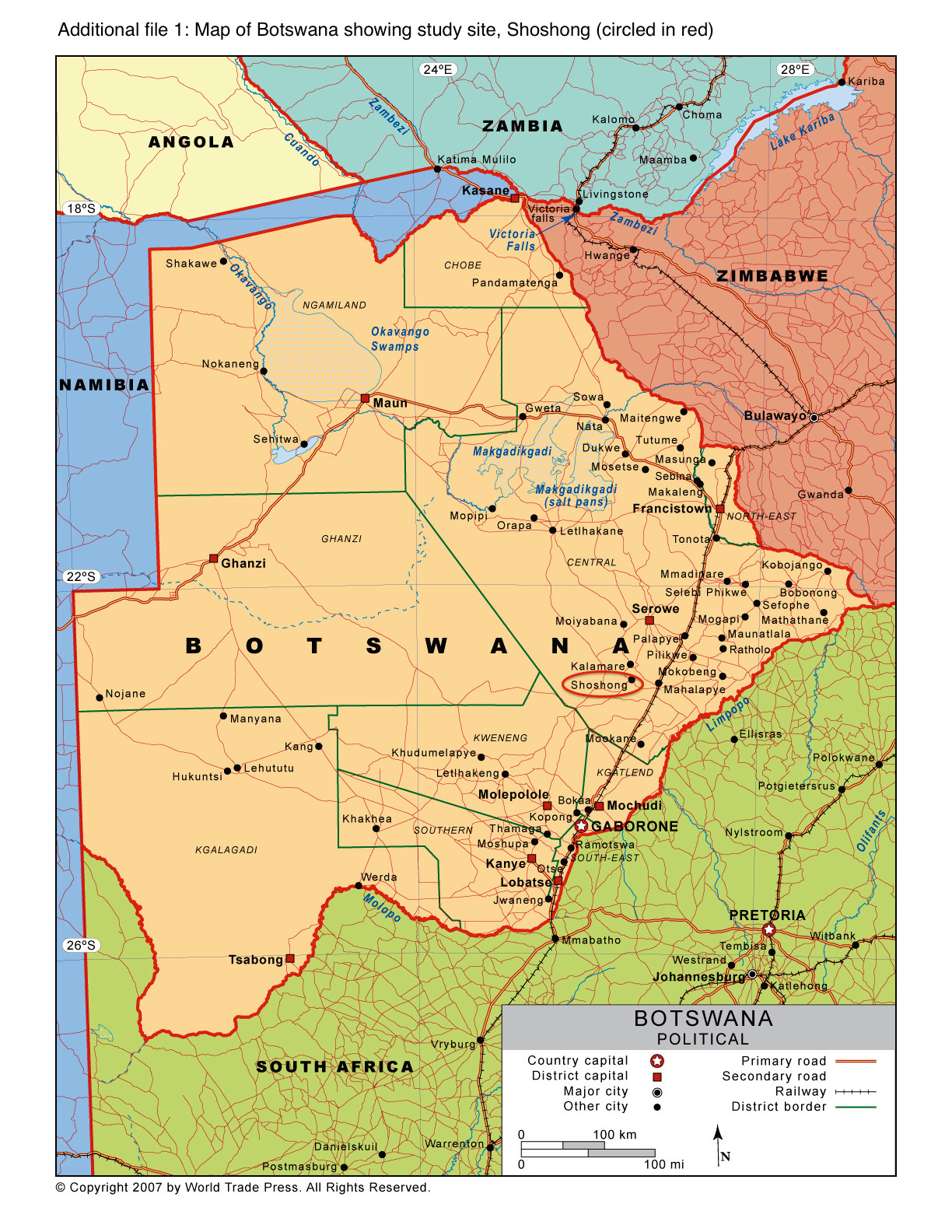

Supplement: Additional file 1: — Map of Botswana showing the study site, Shoshong (circled in red). Royalty free map purchased from World Trade Press© on 27 September 2014. [file 12998_2015_56_MOESM1_ESM.jpeg]
