# Supplementary material for: How can we assess the burden of muscle, bone and joint conditions in rural Botswana: context and methods for the MuBoJo focused ethnography
Source: Chiropr Man Therap. 2015 Mar 16;23:11. doi: 10.1186/s12998-015-0056-9 (PMC4361207; doi:10.1186/s12998-015-0056-9)

Additional file 2: MuBoJo brochure, Setswana

Babatlisisi ba tla a buisanya le baagi ba motse ka tse …

Matshwenyego a gago a letsatsi le letsatsi a mesifa, marapo le ditokololo (MuBoJo).

Ka fa matshwenyego a mesifa, marapo le ditokololo a amang botshelo jwa gago le tiro ka teng.

Ditsela tse di mosola go tlhokomela mathata a mesifa, marapo le ditokololo (MuBoJo).

Dithuso tse di tlhokafalang tsa mathata a mesifa, marapo le ditokololo (MuBoJo) mo Shoshong.

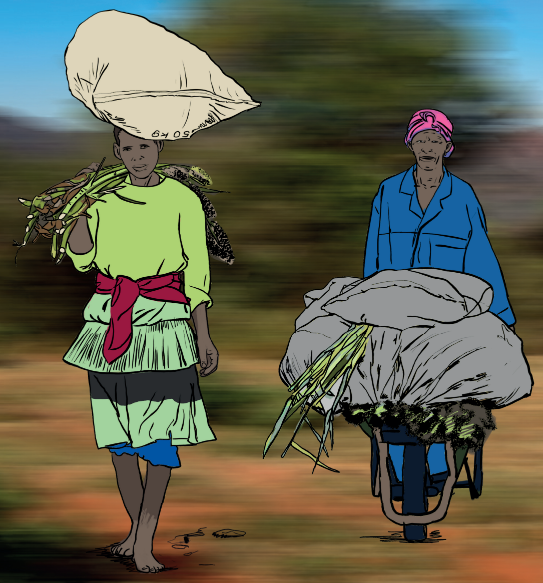

WORLD SPINE CARE

Dipotso ka dipatlisiso?
Moeteledipele wa dipatlisiso
Dr. Maria Hondras
+267 7698 7262

UNIVERSITY OF SOUTHERN DENMARK

Dipotso ka kokelwana ya
World Spine Care
+267 476 9033
(Ka fa marago ga kokelwana ya Shoshong)

Dipotso ka ditshwanelo tsa gago?
Moemedi wa Lephata la Botsogo
Mr. Pilate Khulumani
+267 391 4467

Lephata la Botsogo mo Botswana le letleletse dipatlisiso ka letsatsi la 14 August 2013

PULA
Republic of Botswana

Dipatlisiso Tsa MuBoJo

Ke tsela efe e o ka tshelang le go tlhokomela **Mesifa**, **Marapo** le **Ditokololo** mo **Shoshong?**

Dipatlisiso tse, di tla re thusa go bona ditsela tse ka tsone re ka kgonang go tlhokomela tsa botsogo mo motseng

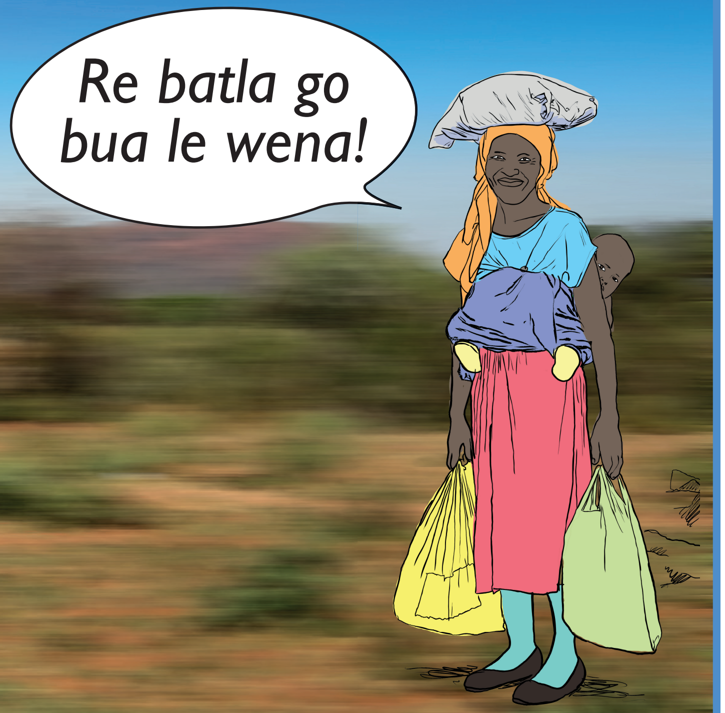

folderSetswana.indd 1 11/09/13 13.04

A o nale le ditlhabi, go opa, kgotsa matshwenyego mo mmeleng wa gago?

Kgotsa go botlhoko go se kae/go le gonnye.

Kgotsa o itse mongwe yo o nang le mathata a mesifa, marapo le ditokololo?

Ikopanye le rona! Re batla go buisanya le borre le bomme ba dingwaga tse di faraloganyeng.

Mu

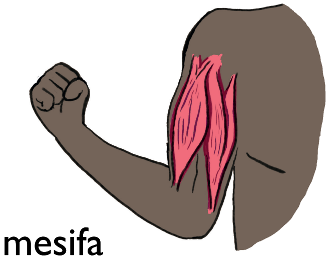

Muscles
(Mesifa)
Mmele o na le mesifa e le makgolo a marataro le masome a matlhano (650).

Bo

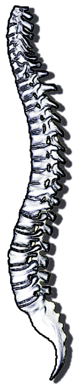

Bones
(Marapo)
Mmele o na le marapo a le makgolo a mabedi le borataro (206).

Jo

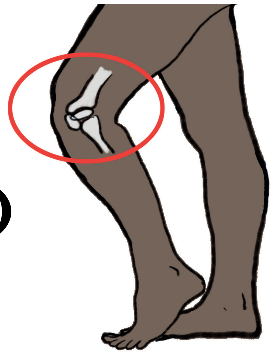

Joints
(Ditokololo)
Mmele o na le ditokololo di le lekgolo, masome a roba bobedi le bosupa (187).

Re batla go ithuta mo go wena!

Dipuisanyo di ka nna tsa tsaya sebaka sa Oura.

Sengwe le sengwe se o se re bolelelang ke sephiri.

Ke tshwetso ya gago go tsenelela dipatlisiso tse.

O gololesegile go emisa dipuisanyo tsa dipatlisiso tse, nako e nngwe le e nngwe kwa ntle ga go fa lebaka.

Re thuse gore re kgone go thusa motse wa Shoshong!

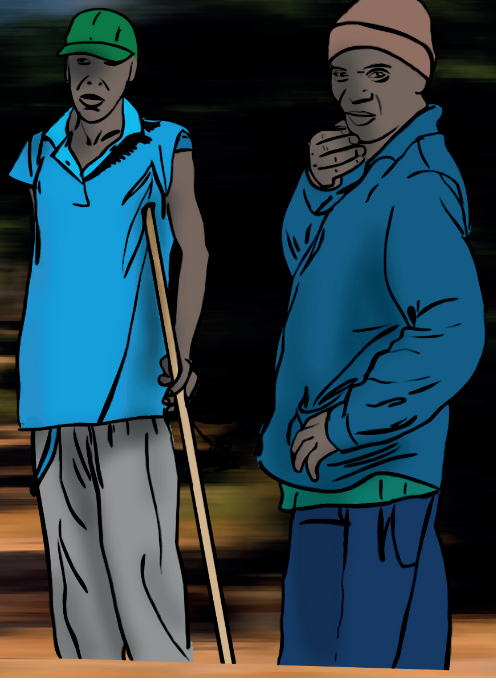

Supplement: Additional file 2: — MuBoJo brochure, Setswana. [file 12998_2015_56_MOESM2_ESM.pdf]
