# Supplementary material for: How can we assess the burden of muscle, bone and joint conditions in rural Botswana: context and methods for the MuBoJo focused ethnography
Source: Chiropr Man Therap. 2015 Mar 16;23:11. doi: 10.1186/s12998-015-0056-9 (PMC4361207; doi:10.1186/s12998-015-0056-9)
Supplement: Additional file 3: — MuBoJo brochure, English. [file 12998_2015_56_MOESM3_ESM.pdf]

Researchers will interview Villagers about…

Your everyday troubles from MuBoJo conditions.

How MuBoJo troubles affect your life and work.

Helpful ways you care for MuBoJo conditions.

Services needed for people with MuBoJo conditions in Shoshong.

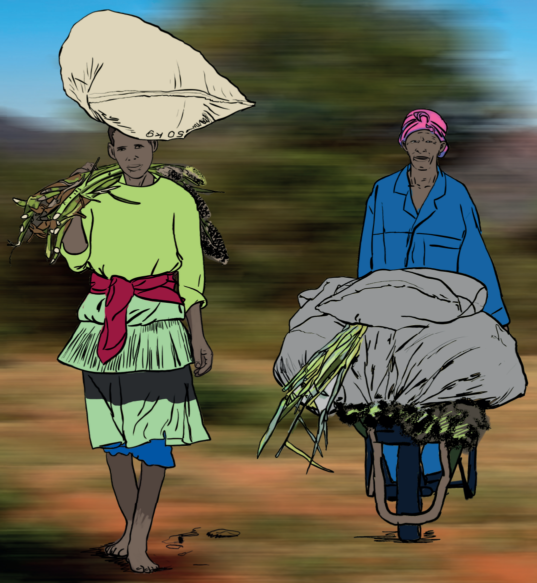

Questions about this project?
Lead Researcher
Dr. Maria Hondras
+267 7698 7262

Questions about
World Spine Care Clinic?
+267 476 9033
(behind the Shoshong Clinic)

Questions about your rights?
MoH Representative
Mr. Pilate Khulumani
+267 391 4467

The Botswana Ministry of Health approved this project on 14 August 2013

MuBoJo Project

What is the best path to live with and care for **Mu**scles, **Bo**nes & **Jo**ints in **Shoshong**?

This project will help us plan
Village healthcare
services

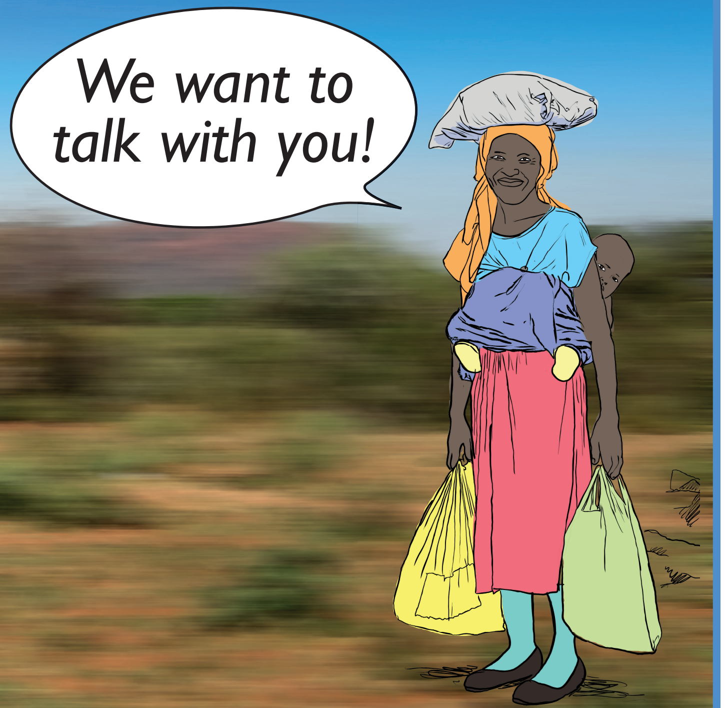

folder1.indd 1 11/09/13 13.04

Do you have aches, pains, or troubles in your body?

Maybe you hurt a little.

Maybe you hurt a lot.

Maybe you know someone with **MuBoJo** problems?

Contact us! We want to talk with men and women of all ages.

Mu

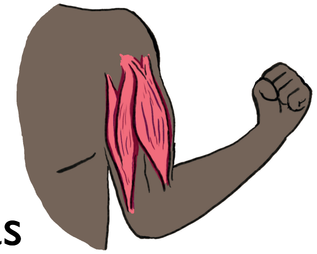

Mesifa
The body has 650 **Mu**scles.

Bo

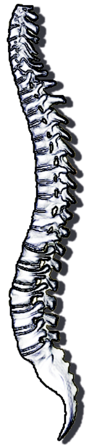

Marapo
The body has 206 **Bo**nes.

Jo

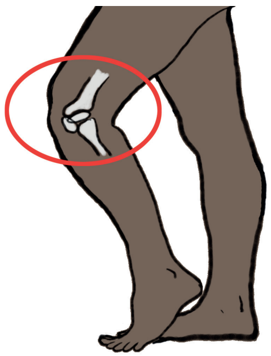

Ditokololo
The body has 187 **Jo**ints.

We want to learn from you!

Interviews may last one hour.

The information you share with us is private.

It is your choice to join this study.

You may stop at any time & do not need a reason.

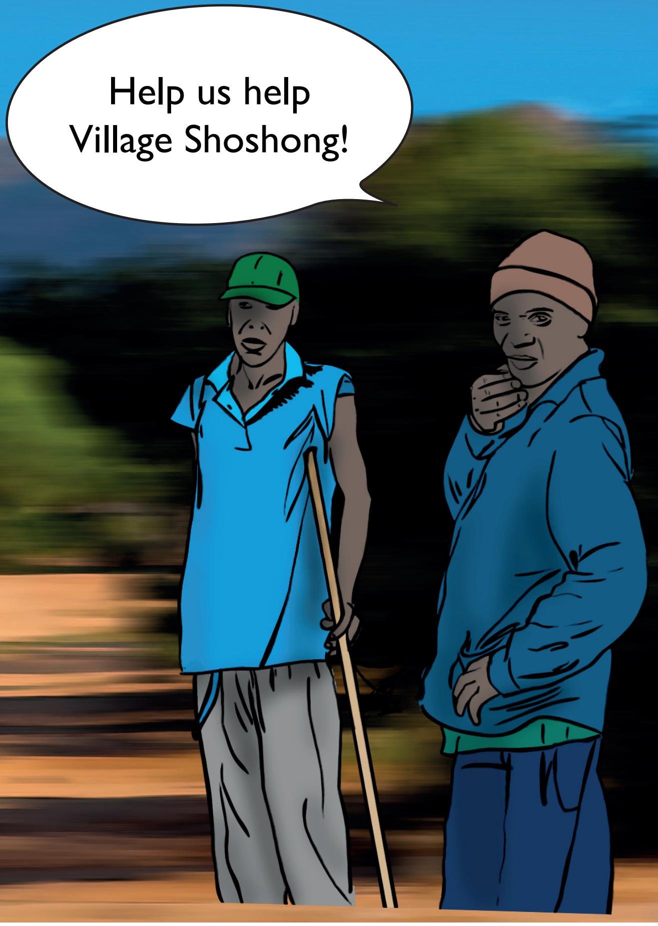

folder1.indd 2 11/09/13 13.04
