# Supplementary material for: How can we assess the burden of muscle, bone and joint conditions in rural Botswana: context and methods for the MuBoJo focused ethnography
Source: Chiropr Man Therap. 2015 Mar 16;23:11. doi: 10.1186/s12998-015-0056-9 (PMC4361207; doi:10.1186/s12998-015-0056-9)
Supplement: Additional file 4: — Informed Consent Document, Setswana. [file 12998_2015_56_MOESM4_ESM.pdf]

Dipatlisiso Tsa MuBoJo
Tumalano ya go Tsenelela Dipatlisiso
Dipuisanyo Tsa Moagi wa Motse le Modiri wa Tsa Botsogo

Setlhogo Sa Dipatlisiso: Mathata a letsatsi le letsatsi a matlhoko a mesifa, marapo le ditokololo **(MuBoJo)** a a amang baagi ba Shoshong botshelo botlhe.

Moeteledipele Wa Dipatlisiso: Maria A. Hondras DC, MPH
World Spine Care Research Team
PhD Student, University of Southern Denmark

Ditlhaloso Ka Dipatlisiso

Re buisanya le baagi ba Shoshong go tlhaloganya gore mathata a mesifa, marapo le ditokololo di ama matshelo a bone jang. Dipuisanyo tsa rona di tla a bo di ntse jaana: Re tla a buisanya le motho a le nosi ka nako kgotsa re buisa setlhopha sa baagi ba motse le ba bereki ba tsa botsogo ba ba nnang, le go tlhokomela batho ba ba tshelang ka matlhoko a mesifa, marapo le ditokololo. Dipatlisiso tse, di tla re thusa go akanyetsa tlhokomelo ya batho ba motse o. Lephata la botsogo mo Botswana, le dumetse gore dipatlisiso tse, di ka dirwa.

Ke Mang Yo O Ka Di Tsenelelang

Baagi ba Shoshong le babereki ba botsogo ba ba dingwaga tse di lesome le borobabobedi ba ka tsenelelela dipatlisiso tse. Motsenelela dipatlisiso tse o tshwanetse go bo a itse go bua puo ya Setswana kgotsa sekgoa go kgona go araba dipotso.

Tsamaiso Ya Dipatlisiso

Ke dipatlisiso tsa tshekatsheko ka kelotlhoko. Mo dipatlisisong tsa tshekatsheko motho ga a dirisiwe e ka re go lekeletswa gore a kalafi e tla a bereka. Motho yo o dirang dipatlisiso o tla a go botsa dipotso tse di amang mathata a botsogo, ditiro tsa letsatsi le letsatsi, mesifa, marapo le ditokololo le tlhokomelo e e fiwang.

Motho yo o rutuntsheditsweng go buisanya le batho, le yo o tolokololang, ba tla a go botsa dipotso mo lwapeng la gago, mo kamoreng ya sephiri kwa kokelwaneng ya Shoshong kgotsa fa go itlhophetsweng ke wena. Dipuisanyo tse di ka nna tsa tsaya sebaka sa oura kgotsa tse pedi. Nako e key a go tlatsa pampiri ya tumalano ya go tsenelela dipatlisiso tse, le go araba dipotso tsa dipatlisiso. Mo gongwe, re ka nna ra ra go kopa go tlhakana le setlhopha sa batho ba bangwe ba motes, kgotsa babereki ba botsogo go buisanya ka go tshela le tlhokomelo ya batho ba matlhoko a mesifa, marapo le ditokololo.

Re ka nna ra dira mokwalo fa puisanyo e ntse e tsweletse. Mokwalo o, ga o na go tsenya leina la gago. Re tla a tsaya mantswe a batho ga dipuisanyo di ntse di tsweletse gore re tle re itirele mokwalo kwa bofelong. Ga re na go dirisa maina a gago ka nako ya go tsaya mantswe le fa re dira mokwalo. Ba batlisisi ke bone fela ba tla a itseng ka dikarabo.

Ditsamaiso Tse Dingwe Tsa Dipatlisiso/Alternative Procedures

Ga gona ditsamaiso tse dingwe tsa dipatlisiso tse.

Ditlhathobo Tsa Madi

Ga gona madi kgotsa dinama tse di tla a kgaolwang mo bathong go dira dipatlisiso tse .

Diphatsa/Matshwenyego

Ga gona diphatsa dipe tse di ka solofelwang mo dipatlisiso tse. Re tla a go botsa dipotso ka mathata a mesifa, marapo le ditokololo, le ka fa batho ba itlhokomelang ka teng go fokotsa mathata a. Batho ba bangwe ba nna le mathata a go araba dipotso tsa mofuta o. O gololesegile go gana go araba dipotso, le go emisa go botswa dipotso ga o batla.

Go Tlhoka Go Ikutlwa Sentle

Fa o ikutlwa o sa tsoga ka nako ya go botswa dipotso, ka ntata ya go tsenelela dipatlisiso tse, o tla a fiwa thuso ka go isiwa kwa kokelong e e tshwanetseng kgotsa go sidilwa maikutlo.

Dipoelo

Ga gona dituelo tse o tla a di fiwang ka go bo o tseneletse dipatlisiso tse. Ka tsela e nngwe o tla a akola go buisanya ka botsogo le bogole jwa gago le bangwe ka wena. Maduo a dipatlisiso tse a tla thusa go tlhaloganya ka fa matlhoko a mesifa, marapo le ditokololo a amang matshelo a letsatsi le letsatsi ka teng. Maduo a a botlhokwa a, a ka thusa go tokafatsa tlhokomelo mo Botswana le lefatshe ka Bophara.

Kitso E Ntsha

Maduo a dipatlisiso tse a masha, a tla a neelwa ba lephata la Botsogo. Fa maduo mangwe a masha a ka dira gore o se ka wa tsenelela dipatlisiso, re tla a go itsise mo ka maduo a ka sebele.

Ditebogo Tsa Ba Tsenelela Dipatlisiso

Ga o na go duelela go tsenelela dipatlisiso tse, gape ga ba na go duelelwa go di tsenelela. Re tsaya tsie dikarabo tse batho ba ba tseneletseng dipatlisiso tse ba re di neelang. Ka jalo go tla a nna le lemmenyana go leboga.Puisanyo e e tla a tsaya sebaka sa oura kgotsa tse pedi. Ga o na go duelelwa go tsenelela dipatlisiso tse. Re tsaya tsiya dikarabo tse o re di neelang o le motsenelela dipatlisiso. Ka jalo, o tla a nna le tshono ya go fiwa kgetsana ya letsela e e sa onaleng ka bonako.

Baithaopi Go Tsenelela Le Go Tlogela Dipatlisiso

Go tsenelela dipatlisiso tse ga go pateletswe, batho ba le tsenelela ka go ithaopa. O gololesegile go itlhophela go seka o tsenelelela dipatlisiso tse. Fa o tseneletse dipatlisiso, o gololesegile go gana go araba potso nngwe le nngwe, le go emisa go nna motseneledi. Ga ba na go otlhaiwa kgotsa go seka ba fiwa malebogo a a ba lebaganeng. Ga o itlhophela go se ka o tsenelela dipatlisiso, kgotsa o emisa go araba dipotso tsa dipatlisiso, ga o na go otlhaiwa kgotsa rontshiwa ditswanelo tsa gago.

Sephiri

Dikarabo tsotlhe tsa gago tse o di re neelang ke sephiri. Dikarabo di ka ntshetswa kwa ntle ka tumalano ya gago kgotsa di batliwa ke ba lephata la Botsogo. Dikarabo tsa motho mongwe le mongwe di tla a fiwa nomore e e pharologanyo.Dikarabo tsotlhe tse dinang le maina a gago di tla a beiwa mo sephiring. Fa go kwalwa maduo a dipatlisiso, ga re na go kwala maina a gago mo go one. Maduo otlhe le dikarabo di tlalotlelelwa mo dikobotong, motho ofe kgotsa ofe ga a na go letlelelwa go bona maduo le dikarabo tsa dipatlisiso, ga e se batho ba ba tshwanetseng. Maduo a a mo dicomputareng, a tla a bo a sireleditswe ka maemo a a kwa godimo.

Tiriso Ya Maduo

Mogolwane wa dipatlisiso o tla a dira gore nomore e o e filweng, e tsamaelane le maina a gago go netefatsa gore re ka kgona go itse ka botsogo le tlhokomelo ya gago mo nakong e e tlang.

Itshwaraganye Le Mang

Ga o nale dipotso ka dipatlisiso tse, o ka itshwaraganya le Dr. Maria Hondras ko +267 7698 7262 or mhondras@health.sdu.dk. Fa o na le dipotso mabapi le ditshwanelo tsa gago jaaka motsenelela dipatlisiso tse, o ka itshwaraganya le Mr. Pilate Khulumani, moemedi wa lephata la Botsogo mo Botswana, nomore ke +267 391 4467.

[NEXT, administer the Statement of Consent.]
