# Supplementary material for: How can we assess the burden of muscle, bone and joint conditions in rural Botswana: context and methods for the MuBoJo focused ethnography
Source: Chiropr Man Therap. 2015 Mar 16;23:11. doi: 10.1186/s12998-015-0056-9 (PMC4361207; doi:10.1186/s12998-015-0056-9)
Supplement: Additional file 5: — Informed Consent Document, English. [file 12998_2015_56_MOESM5_ESM.pdf]

MuBoJo Project
INFORMED CONSENT DOCUMENT
Villager & Healthcare Worker Interviews

Project Title: Everyday problems, lifelong impact, and care of muscle, bone and joint (**MuBoJo**) conditions among Shoshong Villagers

Lead Researcher: Maria A. Hondras, DC, MPH
World Spine Care Research Team
PhD Student, University of Southern Denmark

Project Description

We are interviewing people in Shoshong to understand how muscle, bone and joint troubles impact their lives. We will conduct individual and small group interviews with Villagers and healthcare workers who live with and care for people living with musculoskeletal disorders. This project will help us plan for Village healthcare needs. The Botswana Ministry of Health has approved this study.

Eligibility Criteria

Shoshong Villagers and healthcare workers at least 18 years of age are eligible to join this study. You must speak Setswana or English to answer the interview questions.

Study Procedure

This is an observational research study. An observational study means the researcher does <u>not</u> place you in any experimental treatment group. Rather, the researcher will ask you questions about health, daily activities, muscle, bone and joint problems and the care given for these problems.

A trained interviewer and interpreter will ask you questions during an interview in your home, in a private room at the Shoshong Clinic, or at a place you choose. The interview could take one or two hours to complete. This includes time to complete the informed consent process and participate in the interview. In some cases, we may ask you to join a small group interview with other Villagers or healthcare workers to discuss living with or caring for people living with muscle, bone and joint problems.

We may write notes during the interview. These notes will not include information that identifies you by name. We will audio record the interviews and make transcripts of our talks. We will not use your name in the recording or transcript. Only researchers have access to the data.

Alternative Procedures

There are no alternative procedures to this interview.

Blood Tests

No blood samples or tissue specimens will be collected for this study.

Risks and/or Discomfort

There are no physical risks anticipated in this study. We will ask you questions about musculoskeletal problems and about how you care for these problems. Some people might find it stressful to answer questions about these topics. You can refuse to answer any question and stop participating at any time.

Handling of Research Related Injury

If you experiences distress because of taking part in this study, you will receive an appropriate referral for healthcare or counseling.

Benefits

There may be no direct benefit to you from taking part in this study. On the other hand, you may find satisfaction sharing your experiences and opinion about health and disability. The data from this project will help researchers understand how muscle, bone and joint troubles impact daily life. This valuable information may help improve the care in Botswana and also throughout the world.

New Information

Any new information from this project will be prepared in a report for the Ministry of Health. Should any new information change your decision to participate, we will inform you directly about this information.

Costs to Subjects and Compensation

You will not pay for participating in any part of this study. Completing the interview will take about 1-2 hours of your time. You will not be paid for being in this research study. We value the information you share during the interview and will give you a durable fabric bag as thanks for joining this project.

Voluntary Participation and the Right to Withdraw

Taking part in this research study is voluntary. You may choose not to take part at all. If you decide to take part, you can refuse to answer any question and stop participating at any time. If you decide not to be in this study or stop participating at any time you will not be penalized or lose benefits for which you otherwise qualify.

Privacy, Anonymity and Confidentiality

The information you share during this project will remain confidential and will be disclosed only with your permission or as required by the Ministry of Health. We will assign a unique number to each record in this study. We will keep private all records that identify you by name. If we write a report or article about this study we will not identify individual people. We will store all information in locked cabinets and only authorised researchers will have access to this information. All computer files will be stored on a secure computer and will be password protected.

Future Use of Information

The lead researcher will link your unique number in the secure database system to your name, so that we may follow your health conditions and care over time.

Who to Contact

If you have questions about this study, you may contact Dr. Maria Hondras at +267 7698 7262 or mhondras@health.sdu.dk. If you have questions about your rights as a research participant you may contact Mr. Pilate Khulumani, Botswana Ministry of Health representative at +267 391 4467.

[NEXT, administer the Statement of Consent.]
