# Supplementary material for: How can we assess the burden of muscle, bone and joint conditions in rural Botswana: context and methods for the MuBoJo focused ethnography
Source: Chiropr Man Therap. 2015 Mar 16;23:11. doi: 10.1186/s12998-015-0056-9 (PMC4361207; doi:10.1186/s12998-015-0056-9)
Supplement: Additional file 6: — Statement of Consent, Setswana. [file 12998_2015_56_MOESM6_ESM.pdf]

Dipatlisiso tsa MuBoJo
Dipuisanyo Tsa Moagi wa Motse le Modiri wa Tsa Botsogo

Setlhogo sa Dipatlisiso: Mathata a letsatsi le letsatsi a matlhoko a mesifa, marapo le ditokololo **(MuBoJo)** a a amang baagi ba Shoshong botshelo botlhe.

Moeteledipele wa Dipatlisiso: Maria Hondras DC, MPH
World Spine Care Research Team
PhD Student, University of Southern Denmark

Tlhaloso ka Dipatlisiso: Re buisanya le batho ka go ba botsa dipotso go tlhaloganya gore matlhoko a mesifa, marapo le ditokololo a ama matshelo a baagi ba Shoshong jang? Babatlisisi ba tla a dirisa maduo go thusa ka tlhokomelo ya batho ba matlhoko a mesifa, marapo le ditokololo.

Seemo Sa Tetla: Lephata la Botsogo la Botswana le letleletse dipatlisiso tse.
Letsatsi La Tetlelelo: 14 August 2013

Tumalano ya go Tsenelela Dipatlisiso

Ke tlhaloganya gore ke tshwetso ya me go tsenelela dipatlisiso tse. Ke na le tshwanelo ya go emisa go araba dipotso nako e nngwe le e nngwe ga ke batla, gape ga ke tlamege go fa lebaka la gore ke eng ke emisa dipuisanyo.

Ke tlhaloganya gore ke kgona go lebelela tsamaiso ya dipatlisiso tse nako e nngwe le e nngwe ka go botsa boeteledipele jwa Phaleng, Bokaa le Kgamane Ward (Dikgosi), kgotsa mogolwane wa kokelwana ya Shoshong, kgotsa mookamedi wa dithuto tsa botsogo (Senior Health Education Assistant).

Ke nnile le sebaka sa go botsa dipotso mabapi le dipatlisiso.

Ke dumela go tsenelela dipatlisiso tse tsa MuBoJo.

Letsatsi la _____ kgwedi ya _____________ ngwaga wa 20____

Go Tsewa Lentswe

☐ Ke fa tetla ya go tsewa lentswe la dipatlisiso tse.

☐ Ga ke letlelele go tsewa lentswe la dipatlisiso tse.

______________________________	______________________________
Leina la Motseneledi	Leina la Mosupi
______________________________	______________________________
Setlanyo sa Motseneledi	Setlanyo sa Mosupi
