# Supplementary material for: How can we assess the burden of muscle, bone and joint conditions in rural Botswana: context and methods for the MuBoJo focused ethnography
Source: Chiropr Man Therap. 2015 Mar 16;23:11. doi: 10.1186/s12998-015-0056-9 (PMC4361207; doi:10.1186/s12998-015-0056-9)
Supplement: Additional file 7: — Statement of Consent, English. [file 12998_2015_56_MOESM7_ESM.pdf]

MuBoJo Project
Villager & Healthcare Worker Interviews

Project Title: Everyday problems, lifelong impact, and care of muscle, bone and joint (**MuBoJo**) conditions among Shoshong Villagers

Lead Researcher: Dr. Maria Hondras
World Spine Care Research Team
PhD Student, University of Southern Denmark

Project Description: We are doing interviews to understand how muscle, bone and joint conditions affect the lives of people in Shoshong. Researchers will use the information to help take care of people with these conditions.

Approval Status: The Botswana Ministry of Health has approved this project.
Approval Date: 14 August 2013

STATEMENT OF CONSENT

I understand it is my choice to join this project. I can stop at any time and do not need to give a reason.

I understand that I can review details about this project at any time by asking the Kgamane, Bokaa and Phaleng Ward Chiefs or the Shoshong Clinic Doctor, Head Matron, or Senior Health Education Assistant.

I had the chance to ask questions about the study.

I agree to take part in the MuBoJo Project.

Dated this ______ day of ______________, 20____

Voice Recording

☐ I give my permission to have a voice recording made of this interview.

☐ I DO NOT give my permission to have a voice recording made of this interview.

_________________________ Participant Name (please print)

_________________________ Witness Name (please print)

_________________________ Participant Signature

_________________________ Witness Signature
