# Supplementary material for: How can we assess the burden of muscle, bone and joint conditions in rural Botswana: context and methods for the MuBoJo focused ethnography
Source: Chiropr Man Therap. 2015 Mar 16;23:11. doi: 10.1186/s12998-015-0056-9 (PMC4361207; doi:10.1186/s12998-015-0056-9)
Supplement: Additional file 8: — Photograph permission, Setswana. [file 12998_2015_56_MOESM8_ESM.pdf]

Dipatlisiso Tsa MuBoJo

Tumalano Ya Tiriso Ya Ditshwantsho Tse Di Emeng (Dinepe) Le Tsa Motshikinyego (Videos)

Ke fa World Spine Care tetla ya go dirisa dutshwantsho tsa me tse di tserweng tsa motshikinyego (videos) le tse di emeng (dinepe) gongwe le gongwe fa go lebaneng le mesifa, marapo le ditokololo kwa ntle ga tuelo (Dipatlisiso Tsa MuBoJo).

Ke tswelela ka go dumalana gore ga ke na go tseela World spine Care dikgato dipe mabapi le dingongorego dife fela tse di ka nnang teng kgatlhanong le ditshwantsho tsa go nna jalo, fa di diriseditswe mesifa, marapo le ditokololo jaaka go tlhalositswe.

Letsatsi la ______ kgwedi ya _______________, 20____

______________________________	______________________________
Leina	Leina la Mosupi
______________________________	______________________________
Setlanyo	Setlanyo sa Mosupi

Ke tlhaloganya gore ga ke na le dipotso mabapi le Dipatlisiso Tsa MuBoJo, ke ka leletsa moeteledipele wa dipatlisiso tse, Dr. Maria Hondras mo mogaleng wa +267 7679 2761 ikgolaganya le ene, kgotsa go buisanya le badiri ka ene kwa World Spine Care go ka bua le ene. Fa ke na le dipotso mabapi le ditshwanelo tsa me jaaka mo tseneledi wa dipatlisiso tse, ke ka ikgolaganya le Mr. Pilate Khulumani, moemedi mo lephateng la Botsogo mo Botswana (Ministry Of Health) mogala ke +267 391 4467.

Ke lebogela gore o bo o re file tetla go dirisa ditshwantsho tsa gago tsa motshikinyego (videos) le tse di emeng (dinepe) mo dipatlisisong tse.

Ke go eleletsa masego le matlhogonolo,

Dr. Maria Hondras
World Spine Care Research Team
PhD Student, University of Southern Denmark
Campusvej 55, DK-5230, Odense M, Denmark
