# Supplementary material for: How can we assess the burden of muscle, bone and joint conditions in rural Botswana: context and methods for the MuBoJo focused ethnography
Source: Chiropr Man Therap. 2015 Mar 16;23:11. doi: 10.1186/s12998-015-0056-9 (PMC4361207; doi:10.1186/s12998-015-0056-9)
Supplement: Additional file 9: — Photograph permission, English. [file 12998_2015_56_MOESM9_ESM.pdf]

MuBoJo Project

PHOTO RELEASE CONSENT FORM

I give my permission to World Spine Care to use photographs and video recordings of me, without compensation, for use in scientific presentations or publications related to the muscle, bone and joint (MuBoJo) project.

I further agree to hold World Spine Care free and harmless from all claims arising from use of said photographs and video recordings when used within the scope described above.

Dated this ______ day of ______________, 20____

____________________________	____________________________
Print Name	Witness Print Name
____________________________	____________________________
Signature	Witness Signature

I understand if I have questions about the MuBoJo project, I can contact the lead researcher, Dr. Maria Hondras, at +267 7679 2761 or by talking with staff at the World Spine Care Clinic to contact her. If I have questions about my rights as a research participant, I can contact Mr. Pilate Khulumani, Botswana Ministry of Health representative at +267 391 4467.

Thank you for your allowing us to record your photograph for this research study.

With best wishes

Dr. Maria Hondras
World Spine Care Research Team
PhD Student, University of Southern Denmark
Campusvej 55, DK-5230, Odense M, Denmark
