# Supplementary material for: How can we assess the burden of muscle, bone and joint conditions in rural Botswana: context and methods for the MuBoJo focused ethnography
Source: Chiropr Man Therap. 2015 Mar 16;23:11. doi: 10.1186/s12998-015-0056-9 (PMC4361207; doi:10.1186/s12998-015-0056-9)
Supplement: Additional file 10: — Transcription guide. [file 12998_2015_56_MOESM10_ESM.pdf]

Transcription Guide

The Project Overview is provided on page two of this document.

1. Transcribe interviews in their original language.
2. For transcribed Setswana, the second step is to go back and give a contextual translation into English.
3. In general, you do not need to record all "Emma" or "Erra" or "Eheh" if only used to continue the conversation. But if "Emma" - "Erra" - "Eheh" is followed by more words, then please transcribe.
4. For low quality recordings, it is better for empty spaces to be marked as "[INAUDIBLE]" instead of guessing the content of interview sections.

4.1. **SAMPLE notations for inaudible sections** -- these will alert the researcher to listen to the tape again to clarify meaning.[1]

[CANNOT HEAR] Sounds like: (transcriptionist's best guess).
[VERY LOW VOICE] Sounds like: (transcriptionist's best guess).
[EVERYBODY TALKING AT ONCE, VERY EXCITED, MUCH LAUGHING, CANNOT MAKE OUT WHAT IS BEING SAID.]
[SEEMS LIKE SARCASTIC TONE OF VOICE, MEANING THE OPPOSITE OF WHAT IS SPOKEN]
[INTERRUPTION]
[POOR TAPE QUALITY]
[TOO MUCH BACKGROUND NOISE]

4.2. **SAMPLE notations of emotional content** -- use cautiously and do not overuse. These notations will assist the researcher to better understand interview content. [1]

[SOUNDING TEARFUL]
[SNIFFLING]
[WEEPING]
[QUIET WEEPING]
[WEEPING VERY LOUDLY, VERY DISTRESSED]
[LAUGHING AND CRYING]
[LAUGHTER]
[SOUNDS LIKE NERVOUS LAUGHTER]
[QUIETLY]
[LONG PAUSE]
[ANIMATED]
[SUBDUED]
[SPEAKING VERY QUICKLY]
[SOUNDS VERY DISTRESSED TO ME]

5. Common terms, abbreviations & acronyms. Feel free to assign your own by listing at the beginning of the transcript. Here are a few examples you will see for transcribed English:

b/c	because
MAH	Maria A Hondras, researcher/interviewer
MoH	Ministry of Health
MuBoJo	**Mu**scle, **Bo**ne, and **Jo**int project or conditions or health
Pxxx	Participant identification number (e.g. P007)
WSC	World Spine Care

6. If material is sensitive or emotional, please contact the researcher straightaway and schedule a time to discuss concerns.

[1]MacLean LM, Meyer M, Estable A. Improving accuracy of transcripts in qualitative research. *Qual Health Res* 2004;14(1):113-23.

MuBoJo Project

Burden and care of muscle, bone and joint (MuBoJo) conditions among Villagers in Shoshong, Botswana

Project Overview

The broad aim of this focused ethnography is to examine the cultural, social, organisational and clinical characteristics for the burden of living with and caring for people living with musculoskeletal conditions in a rural, developing country setting - Village Shoshong, Botswana.

The specific aims are to:

1. explicate the burden of muscle, bone and joint conditions in Villagers' everyday lives and their community;
2. analyse MuBoJo care behaviours and beliefs among Shoshong Villagers and families; and,
3. explore patient and provider experiences with MuBoJo care, including the bodily, emotional, and contextual experiences during manual therapy encounters; and

These data will inform World Spine Care initiatives in Botswana where we partner with community members to develop research, educational and care pathways delivered by Villagers for Villagers.
